# Screening saikosaponin d (SSd)-producing endophytic fungi from *Bupleurum scorzonerifolium* Willd

**DOI:** 10.1007/s11274-022-03434-x

**Published:** 2022-10-25

**Authors:** Yupeng Cheng, Guangjie Liu, Zhongmeng Li, Yongqiang Zhou, Ning Gao

**Affiliations:** 1 Key Laboratory of Basic and Application Research of Beiyao (Heilongjiang University of Chinese Medicine), Ministry of Education, Harbin, China; 2 Guizhou University of Traditional Chinese Medicine, Guiyang, China; 3 Department of Pharmacy, Heilongjiang University of Chinese Medicine, Heping Road 24, Harbin, 150040 Heilongjiang China

**Keywords:** *Bupleurum scorzonerifolium* Willd., Endophytic fungi, UPLC/Q-TOF-MS analysis, Saikosaponin d, ITS sequence analysis, TEF-1α sequence analysis

## Abstract

Saikosaponin d (SSd) is an important bioactive compound of traditional Chinese medicinal plant *Bupleurum scorzonerifolium* Willd. and exhibits many effects, such as anti-tumor, anti-inflammation and immunomodulatory. Since endophytic fungi possess the natural capacity to produce the similar secondary metabolite to that of their host plants, they are promising as alternative sources of plant bioactive natural products. In this study, in order to search for SSd-producing strains, endophytes were isolated from *B. scorzonerifolium* and were authenticated by the ITS sequence and the translation elongation factor-1alpha gene (TEF-1α) sequence analysis. The profile of metabolites present in the crude exacts was carried out by ultra performance liquid chromatography time-of-flight mass spectrometry (UPLC/Q-TOF-MS) analysis. The results showed that two strains, CHS_2_ and CHS_3_ from *B. scorzonerifolium* could produce SSd by UPLC/Q-TOF-MS analysis, and the amount of SSd produced by strain CHS_2_ and CHS_3_ were about 2.17 and 2.40 µg/mL, respectively. CHS_2_ and CHS_3_ showed a close phylogenetic relationship to *Fusarium oxysporum* and *Fusarium acuminatum*, respectively. According to our concern, no endophytic fungi capable of producing SSd from *B. scorzonerifolium* have been found before. Our clear intention was to isolate and identify these endophytic fungi that produce important active secondary metabolites, and then study the strains that produce this compound on a large scale through fermentation or even genetic study, to provide a feasible and more convenient way for the production of SSd.

**Supplementary Information:**

The online version contains supplementary material available at 10.1007/s11274-022-03434-x.

## Introduction

Epiphytic and endophytic are group of microorganisms that live in healthy host plants (Santamaría and Bayman 2005). Endophytes generally exist in various tissues and organs inside the host plants as least a part of their life cycle without directly causing any obvious external diseases and adverse results (Stone et al. 2000; Quilliam and Jones 2010). It is known that endophytes are beneficial to hosts by protecting plants against a variety of biotic and abiotic stresses (Yan et al. 2019; Abeer et al. 2016; Bian et al. 2021). Endophytic fungi possess the natural capacity to produce and accumulate secondary metabolites. These secondary metabolites have various biological activities and can be directly or indirectly used as medicine to treat different diseases (Aly et al. 2010; Nisa et al. 2015; Kusari and Spiteller 2012; Staniek et al. 2008). In recent years, the metabolites from endophytic microorganisms have shown that endophytes have been valued as potential sources of anti-cancer, antioxidant, antibiotics and immunosuppressive bioactive compounds (Kamel et al. 2020). Endophytic fungi are regarded as alternative resources of medicinal plants because they can produce the same bioactive compounds as their host plant, such as alkaloids, terpenoids, steroids and phenols.

*Nan-Chai-Hu*, also named Radix Bupleuri, the root of *Bupleurum scorzonerifolium* Willd. (Umbelliferae), is a traditional Chinese herb which is used in treating of tumor, allergy and inflammation in Asia (Ashour et al. 2009; The Pharmacopoeia Commission of the PRC 2015). This plant’s secondary metabolites might offer us with a variety of active compounds containing flavonoids, essential oils, coumarins, lignans, triterpene saponins, polyacetylenes and alkaloids. These bioactive components are crucial in disease treatments (Ashour and Wink 2011). Among them, saikosaponins (SSs) were found to be the most active compounds, especially saikosaponin d (SSd), which exhibits many effects, such as anti-tumor, anti-inflammation and immunomodulatory (Zhou et al. 2021; Li et al. 2018). In our previous work, the endophytic fungi from *B. scorzonerifolium* were isolated and the distribution characteristics of endophytes in *B. scorzonerifolium* have been investigated (Gao et al. 2012).

The central goal of this study was to screen the endophytic fungi with the capability of SSd producing from *B. scorzonerifolium*. Ultra performance liquid chromatography time-of-flight mass spectrometry (UPLC/Q-TOF-MS) analysis was performed to detect whether the endophytes isolated from *B. scorzonerifolium* could produce the similar compounds to host plants. Up to now, no endophytic fungi of SSd producing from *B. scorzonerifolium* have been reported.

## Materials and methods

### Isolation of endophytes from ***B. scorzonerifolium***

Endophytic fungi were isolated from different parts (roots, stems, leaves and flowers) of *B. scorzonerifolium* with aseptic operation. For surface sterilization, each part of the plant was washed with sterile water, followed by soaking in 75% ethanol (v/v) for 3 min, rinsing twice with sterile water, soaking in 0.1% mercuric chloride (v/v) for 5 min, and rinsing with sterile water for three times. 5 × 5 mm of segments cut from the treated plant material were cultured on potato dextrose agar (PDA) medium (1 L PDA medium containing potato 200 g, dextrose 20 g and agar 12 g) at 25 °C, checking the growth of endophytic fungi in plant segments every day.

### Ethanol extraction from endophytic fungi

The endophytic fungi were cultured in potato dextrose broth (PDB) medium (1 L PDB medium containing potato 200 g and dextrose 20 g) at 28 °C and shaking at 160 rpm for 10 days in the dark. The cultured fungi were collected by vacuum filtration, and then grinded into powder in liquid nitrogen. Ethanol extraction from the fungal powder with 70% ethanol was evaporated with a rotary evaporator (Eyela, Jpn). The extractions were prepared for SSd analysis by UPLC/Q-TOF-MS.

### SSd analysis by UPLC/Q-TOF-MS

The Waters ACQUITY™ UPLC system (Waters Corporation, MA, USA) was used for UPLC–MS analysis of the samples. The C_18_ reversed-phase column (ACQUITY UPLC™ BEH, 50 mm × 2.1 mm, i.d., 1.7 μm) was used and kept at 40 °C. The liquid chromatography was equipped a 190–400 nm detector. 4 µL of sample was injected. The flow rate was set to 400 µL/min and gradient mobile phase (solvent A was 0.1% formic acid in demonized water, solvent B was 0.1% formic acid in acetonitrile) which was programmed as follows: 0 min, 2% B; 1.0 min, 10% B; 4.0 min, 10% B; 12.0 min, 70% B; 14.0 min, 98% B; 15.0 min, 2% B and kept at 2% B for 2 min, given a total running time of 17 min.

The sample is analyzed by Water Xevo quadrupole time-of-flight mass spectrometer (MS; Manchester, UK), and its metabolic spectrum needs to be collected by an electrospray ionization (ESI) source. According to the preliminary experiment of the system determination, the spectral determination was carried out in the positive ion mode, and the optimum parameters are as follows: desolvation temperature of 350 °C, source temperature of 120 °C, cone voltage of 25 V and capillary voltage of 3.0 kV. Nitrogen with flow rate of 650 L/h and 50 L/h was used as nitrogen and conical gas respectively. The data acquisition rate of the instrument was adjusted to 0.2 s and the scanning interval delay is 0.02 s. The scanning range was 100–1000 m/z. In order to ensure the accuracy and reproducibility of the data, all analyses were done through the use of locking spray. Leucine–enkephalin was used as the positive ion model of acetonitrile (0.1% formic acid):H_2_O (0.1% formic acid) with a concentration of 400 pg/mL ([M + H] ^+^ = 556.2771). The Pareto mode was used to collect the data, and the frequency of phase-locked spray was set to 1 s. Before using the Mass Lynx 4.1 software to process the data, the average scanning of the phase-locked quality data was more than 10 times for correction.

### DNA extraction and fungal identification

The mycelia of the two strains were inoculated into the flask containing 200 mL of PDB medium, and the mycelium biomass of about 100 mg was harvested after incubation on the shaker (160 rpm, 28 °C, 3 days). According to the research of Guo et al. (2001), the genomic DNA of endophytic fungi was isolated and extracted by the CTAB method.

Fungal identification was based on their ITS sequences and TEF-1α sequences, which was accomplished by PCR amplification with the universal primers (V9D 5′-TTA AGT CCC TGC CCT TTG TA-3′; LS266 5′-GCA TTC CCA AAC AAC TCG ACT C-3′) and TEF-1α primers (TEF1-728 F 5′-CAT CGA GAA GTT CGA GAA GG-3′; TEF1-rev 5′-GCC ATC CTT GGA GAT ACC AGC-3′) (Van Den Gerrits and Hoog 1999; Carbone and Kohn 1999). Reaction volumes of 50 µL contained 1 mM dNTPs, 1.75 units of Taq DNA polymerase, 0.2 µg of genomic DNA, 1.5 mM MgCl_2_, 5 µL 10 × PCR buffer and 20 pM of each primer. The mixed samples were amplified in Bio-RadT100TM Thermal Cycler (Bio-Rad, USA) to obtain PCR products. The 5 µl PCR product was verified by electrophoresis display on 1% (w/v) agarose gel in 1× TAE buffer (1 mmoL/L EDTA, 40 mmoL/L Tris, pH 8.0). Then the same primers were used for sequencing (Shanghai Sangon Biologic Engineering Technology and Service Co., Ltd., Shanghai, People's Republic of China).

### Phylogenetic analysis

The BLAST algorithm was used to compare the corresponding ITS sequence and TEF-1α sequence of each strains with the NCBI (National Center for Biotechnology Information) data set on GenBank (NCBI; http://www.ncbi.nlm.nih.gov). The DNAMAN program was used to perform multiple sequence alignments. Molecular evolutionary analysis and the phylogenetic tree construction were conducted using neighbor-joining method by MEGA version 11.0 (Kumar et al. 2008).

## Results

**Fig. 1:**
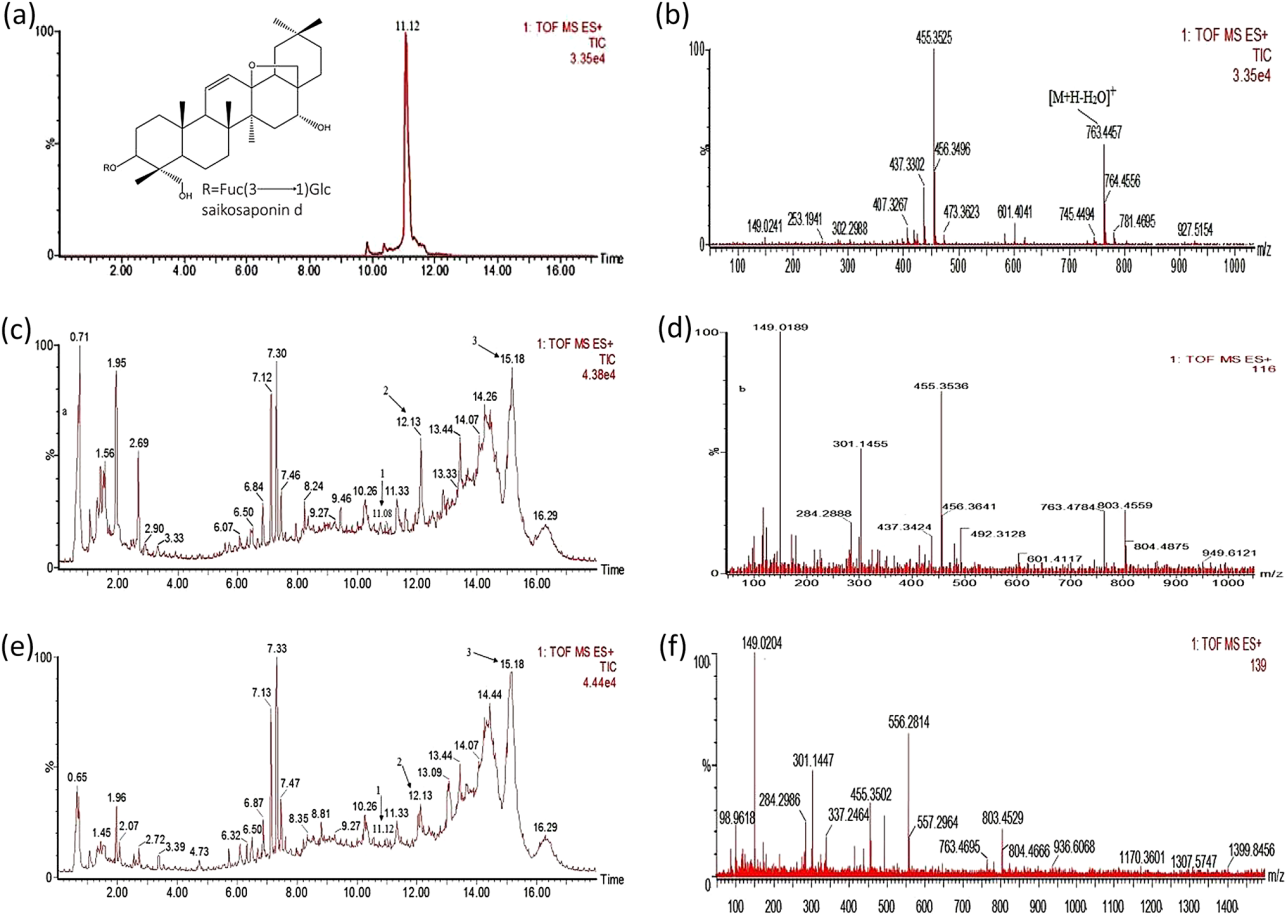
UPLC/Q-TOF-MS analysis of saikosaponin d standard product and metabolites of CHS_3_ and CHS_2_. Total ion chromatogram of standard, CHS_3_ and CHS_2_ metabolites in positive mode (**a**, **c** and **e**) and ES/MS (+) spectra of standard, CHS_3_ and CHS_2_ metabolites from m/z 100 to 1000 (**b**, **d** and **f**)

To assay endophytic fungal metabolites with a reasonable elution time, isocratic elution of acetonitrile–water was performed as described in the experimental section. In our work, the metabolites of CHS_3_ and CHS_2_ (isolated from the stems of *B. scorzonerifolium*) with a running time of 17 min showed the same peaks as those of the SSd standards when eluted at 11.08 and 11.12 min, respectively. The metabolites of CHS_2_ have the same cleavage fragments as the SSD standard at m/z 149.0204, 455.3502, 763.4695 (Fig. 1). The metabolites of CHS_3_ regularly show several fragments at m/z 149.0189, 437.3424, 455.3536, 601.4117 and 763.4784, which are the same as the cleavage fragments of SSd standard (Fig. 1). The amount of SSd produced by CHS_2_ and CHS_3_ were 2.17 and 2.40 µg/mL under the condition described in this study. After subculture, the two endophytic fungi still had a stable ability to produce SSd, and the strains were stored in degree cryogenic refrigerator at − 80 °C.

**Fig. 2:**
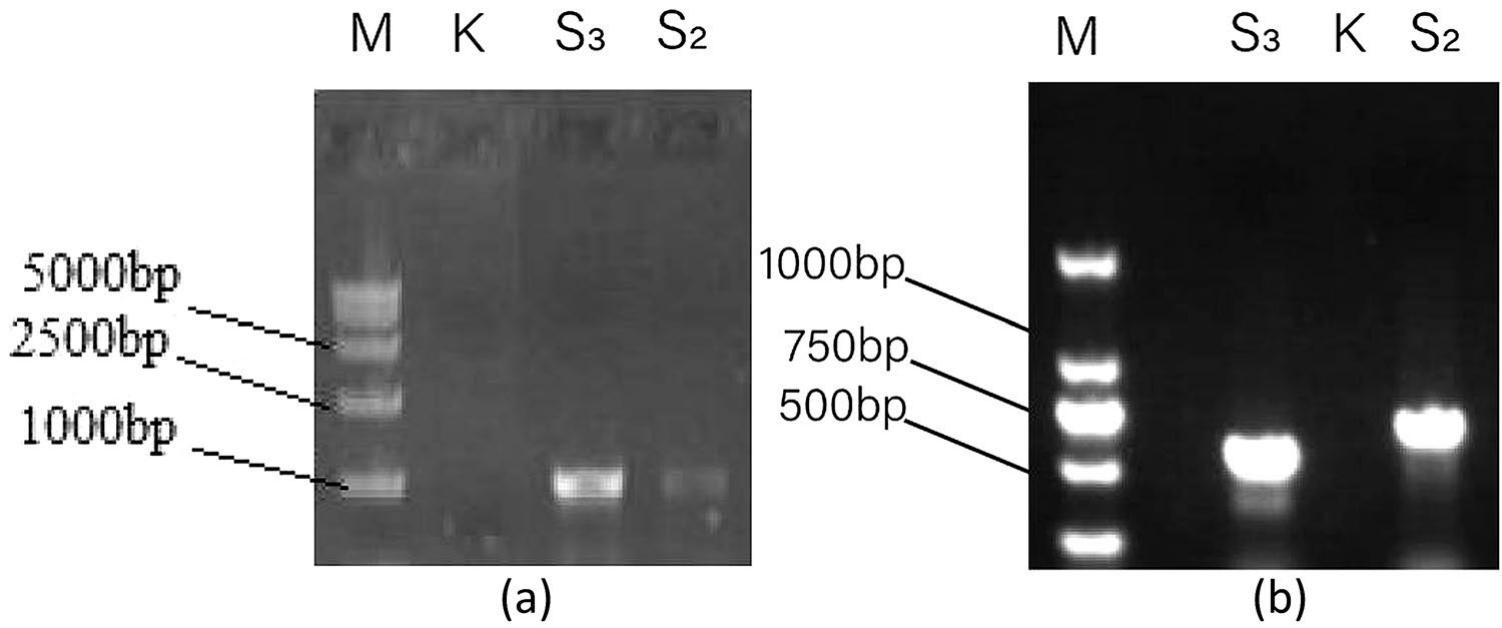
The amplification products of endophytic fungi rDNA from *B. scorzonerifolium * (ITS sequence: **a**, TEF-1α sequence: **b**) (*M* marker, *K* blank)

To further determine the phylogeny of strain CHS_2_ and CHS_3_, the ITS1-5.8 S-ITS2 sequences and TEF-1α sequence of these isolates were amplified and sequenced (Fig. 2). Then the sequences were compared to corresponding sequences of referenced fungal taxa in the database.

**Fig. 3:**
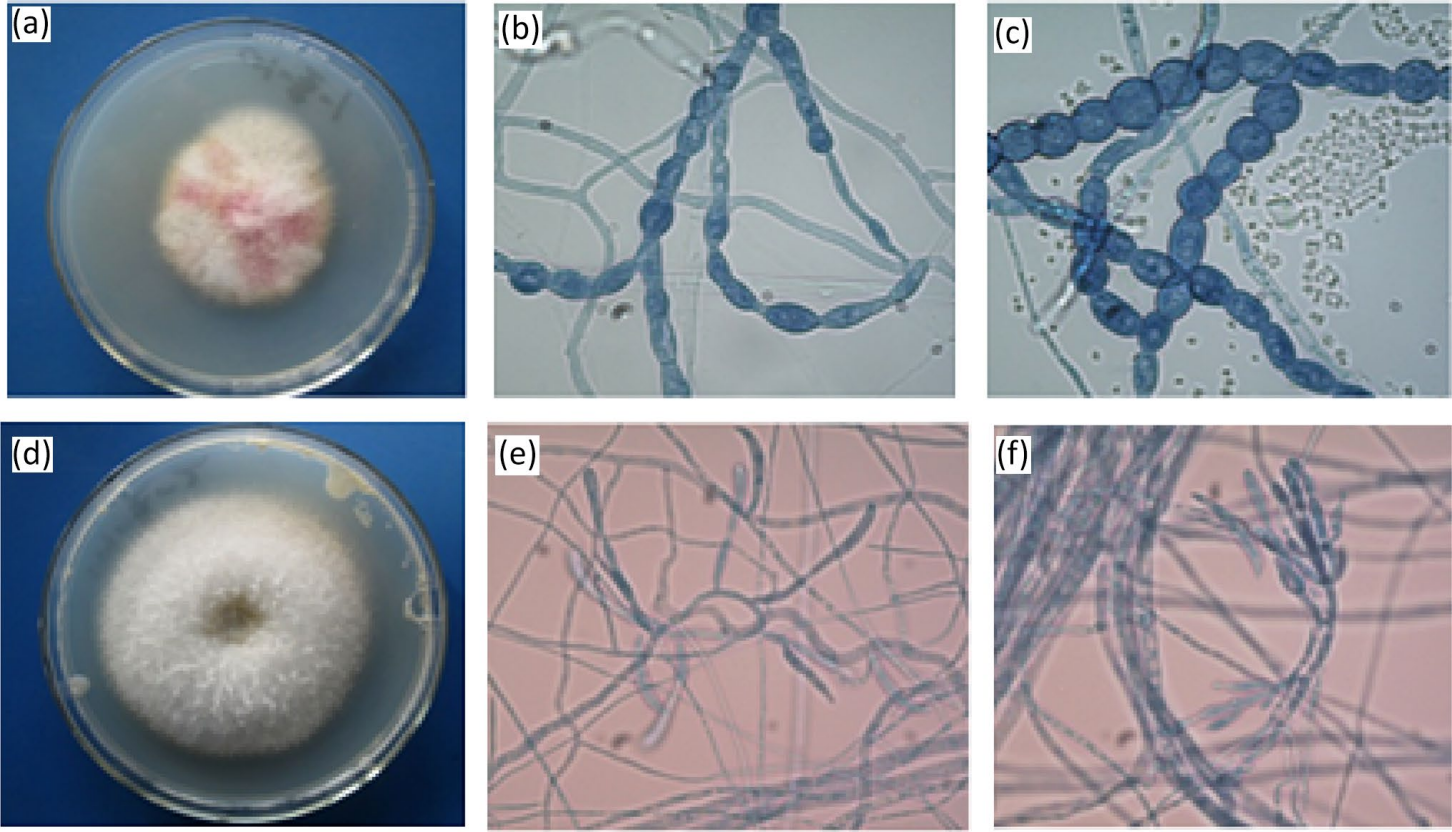
Colony morphology of CHS_2_ and CHS_3_ (**a** and **d**). Mycelia morphology of CHS_2_ and CHS_3_ (**b** and **e**). Conidia morphology of CHS_2_ and CHS_3 _(**c** and **f**)

Also, these fungi were identified by morphological characteristics (Fig. 3). CHS_2_ grew rapidly in PDA medium, the colony was nearly round, the edge was neat, the colony was reddish in the middle, the edge was white, and the texture was loose and fluffy. According to the observation of mycelial microscope, the conidium was irregular and the conidia were chain-shaped. According to the morphological characteristics, CHS_2_ was preliminarily identified as *Fusarium *sp. (Fig. 3). CHS_3_ grew rapidly on PDA medium, the colony was round and uniform, and the hyphae were white, fluffy and well developed. Under microscope, the branches of conidiophores were irregular and distributed in groups. Thick-walled spores are formed in the peduncle of hyphae and cysts. According to the morphological characteristics, CHS_3_ was preliminarily identified as *Fusarium *sp. (Fig. 3).

**Fig. 4:**
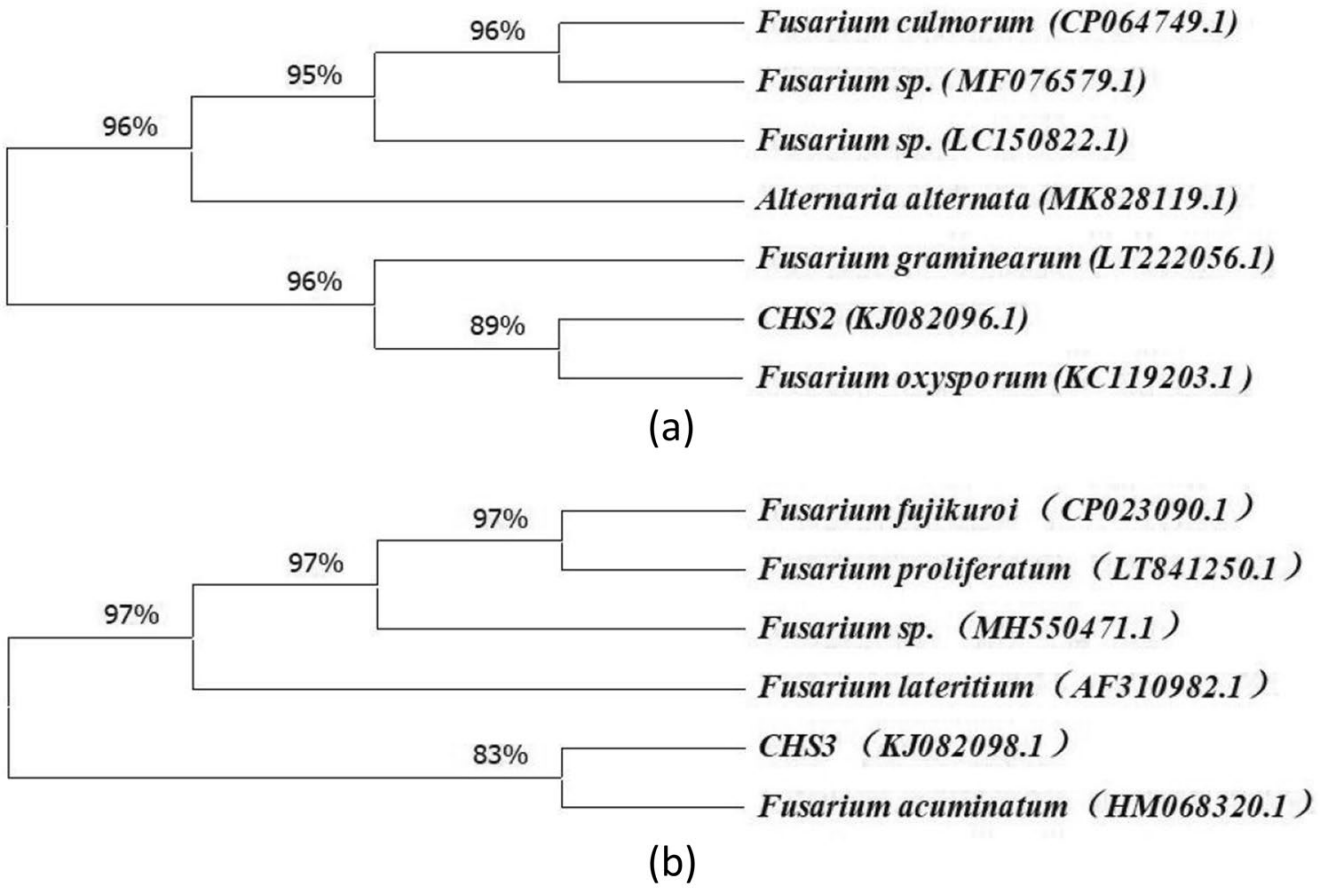
Based on the corresponding ITS sequence of each strain, a strict consensus tree reconstructed by maximum parsimony analysis inferred from the nearest neighbours of endophytic fungi isolated from *B. scorzonerifolium*

**Fig. 5:**
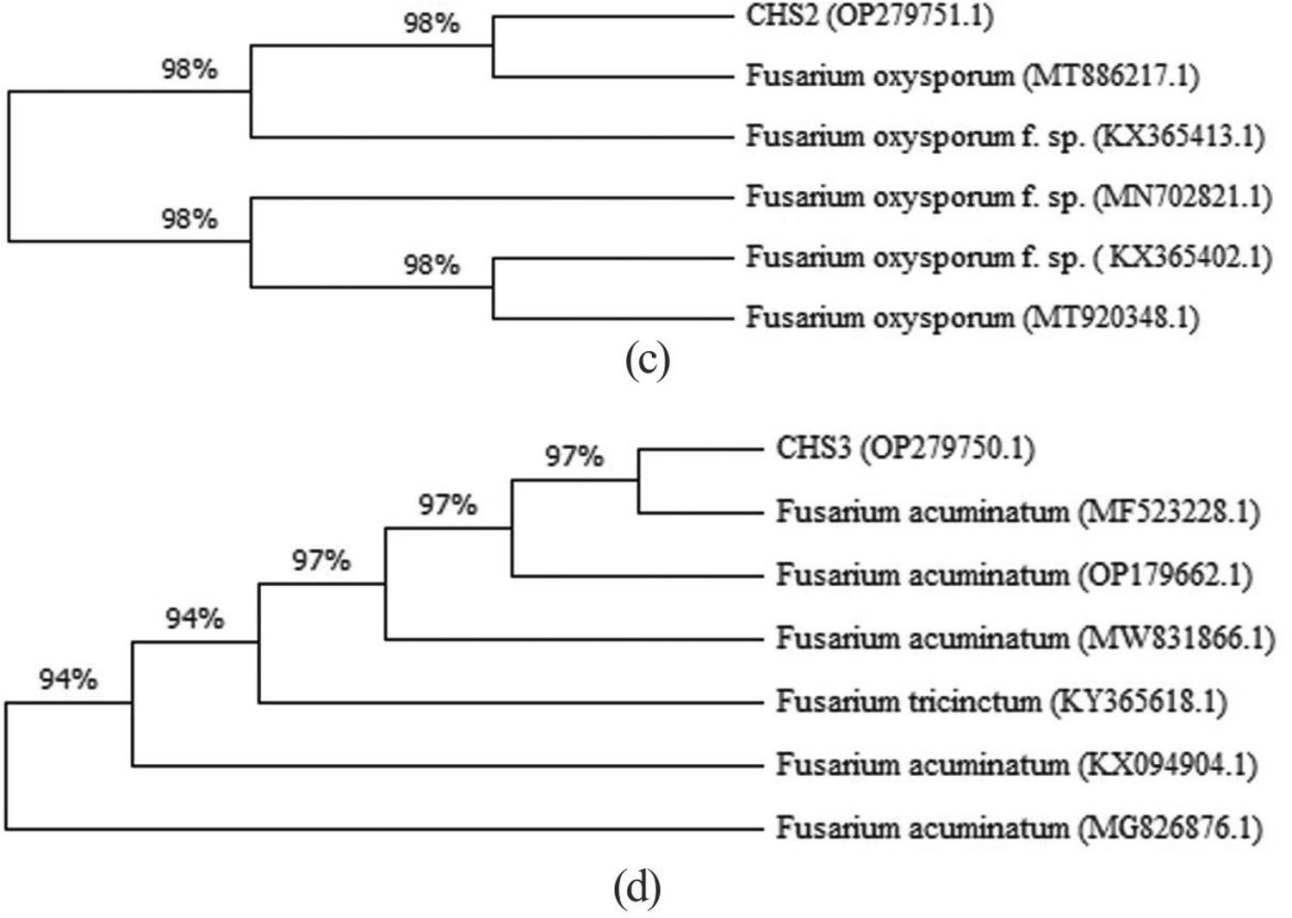
Based on the corresponding TEF-1α sequence of each strain, a strict consensus tree reconstructed by maximum parsimony analysis inferred from the nearest neighbours of endophytic fungi isolated from *B. scorzonerifolium*

Based on ITS sequence and TEF-1α sequence, homologous sequence search with GenBank showed that the similarity between CHS_2_ sequence and *Fusarium oxysporum* was 95% and 98% (GenBank accession numbers KC119203.1 and MT886217.1), and that between CHS_3_ sequence and *Fusarium acuminatum* was 99% and 97% (GenBank accession numbers HM068320.1 and MF523228.1) (Table 1). The phylogenetic relationship was established by comparing and branching the homologous nucleotide sequences of CHS_2_ and CHS_3_ in GenBank. According to the phylogenetic analysis, the isolates of CHS_2_ and CHS_3_ were classified as *F. oxysporum* and *F. acuminatum*, respectively (Figs. 4, 5).

**Table 1:** Identification of endophytic fungi isolated from *B. scorzonerifolium*

Endophytic fungi code	Sequences	GenBank accession number	Identified species	Identities (%)
CHS_2_	ITS	KJ082096	*Fusarium* *oxysporum* KC119203	95
	TEF-1α	OP279751	*Fusarium* *oxysporum* MT886217	98
CHS_3_	ITS	KJ082098	*Fusarium acuminatum* HM068320	99
	TEF-1α	OP279750	*Fusarium acuminatum* MF523228	97

Identities were obtained by blasting sequences in NCBI

## Discussion

Like host plants, endophytic fungi can also possess the natural capacity to produce bioactive secondary metabolites with the same or similar chemical structures (Lamshöft et al. 2008; Cui et al. 2012; Silvia et al. 2007). Therefore, endophytic fungi are considered as potential microbial resources to replace traditional medicinal plants to produce pharmacologically active natural compounds, and have very broad medicinal value and research prospects (Aly et al. 2011). Traditional medicinal plants are the top-priority for isolation and screening of endophytic fungi (Gómez and Luiz 2018). The discovery of SSd-producing endophytic fungi is valuable for industrial application. The production of SSd by endophytes CHS_2_ and CHS_3_ further supports the theory that through long-term symbiotic relationships between endophytic fungi and plants, endophytes may acquire genes from their host plants and could synthesize analogous or identical bioactive secondary metabolites to their hosts. This indicated that CHS_3_ and CHS_2_ could be substituted for *B. scorzonerifolium* to produce SSd in the fermentation industry.

The genus *Fusarium* is widely distributed and is an important source of secondary metabolites, such as camptothecin and vincristine, so it has been widely concerned by people and has become the focus of research in the last few years (Kusari et al. 2009a, b). According to our concern, the genus *Fusarium* has not been found to have the ability to produce SSd before. In this paper, we first reported two SSd-producing endophytic fungi isolated from *B. scorzonerifolium.*

However, the SSd production by each of the two endophytes in axenic cultures is too low to match expectation. Because endophytes always interact with hosts and other endophytes, the biosynthesis ability of endophytic fungi in aseptic culture is very different from that in host plants. When observed metabolic production of the endophytic fungus isolated from *Camptotheca acuminate*, through the first to the seventh-generation subculture, Kusari et al. found that a sharp attenuation occurred in the production of camptothecin by this endophyte (Kusari et al. 2009a, b). They believe that the lack of host stimulation in aseptic cultures may be one of the reasons for the attenuation. It is highly probable that there might be metabolic communication between the endophytes and the host plants, and in vivo metabolic processes of endophytes are likely regulated by plants. Though the mechanisms by which endophytes interact with host plants are incompletely understood, we agreed with the theory that in the process of evolution, endophytic fungi have developed mechanisms for biosynthesis and tolerance to high levels of secondary metabolites in order to better compete and survive with medicinal plants (Kusari et al. 2012). The study of interspecific crosstalk between endophyte–endophyte and endophyte–host is worthy of further study. We have established endophytic–endophytic and endophytic–host co-culture models, and are currently studying the ways in which fungi and hosts interact to promote SSd production.

## Conclusion

This is the first time that we have screened two endophytic fungi with stable genetic characters and the ability to produce SSd. CHS_2_ and CHS_3_ were identified as *F. oxysporum* and *F. acuminatum* respectively by ITS sequence and TEF-1α sequence analysis. All these efforts further prove the natural ability of endophytes to produce the same or similar bioactive substances as host plants, and provide a reliable and stable source for such secondary metabolites in the future.

## Supplementary Information

Below is the link to the electronic supplementary material.

Supplementary material 1 (PDF 386 kb)

## Data Availability

All data generated or analysed during this study are included in this published article (and its Supplementary Information files).
