# Supplementary material for: Screening saikosaponin d (SSd)-producing endophytic fungi from Bupleurum scorzonerifolium Willd
Source: World J Microbiol Biotechnol. 2022 Oct 25;38(12):242. doi: 10.1007/s11274-022-03434-x (PMC9592640; doi:10.1007/s11274-022-03434-x)
Supplement: Supplementary file 1 — Supplementary material 1 (PDF 386 kb) [file 11274_2022_3434_MOESM1_ESM.pdf]

World Journal of Microbiology & Biotechnology

Screening saikosaponin d (SSd)-producing endophytic fungi from *Bupleurum scorzonerifolium* Willd

Yupeng Cheng[1+], Guangjie Liu[1+], Zhongmeng Li[1+], Yongqiang Zhou[2+], Ning Gao[1]*,

[1]Key Laboratory of Basic and Application Research of Beiyao (Heilongjiang University of Chinese Medicine), Ministry of Education

[2]Guizhou University of Traditional Chinese Medicine

[+]Authors contributed equally

***Corresponding author:**

Ning Gao

Department of pharmacy, Heilongjiang University of Chinese Medicine

Heping Road 24, Harbin, Heilongjiang, China, 150040

Tel: 86-0451-87266869

E-mails: yupengcheng617@hotmail.com, guangjieliu2022@163.com, 1157052242@qq.com, Zhouxiaoqiang1988@163.com, gaoning@hljucm.net,

CHS_2 ITS sequence:

TGTAGACAATCTGAGATTTGAGCATCCACCAGACGGGGCTCTCACCCTCTATGGCGTCC
CGTTCCAGGGAACTCGGAAGGCACCGCATCAAAAGCATCCTCTACAAATTACAACTCGGGC
CCGAGAGCCAGATTTCAAATTTGAGCTGTTGCCGCTTCACTCGCCGTTACTAGGGCAATCCC
TGTTGGTTTCTTTTCCTCCGCTTATTGATATGCTTAAGTTCAGCGGGTATTCCTACCTGATCCG
AGGTCAACATTCAGAAGTTGGGGGTTTAACGGCTTGGCCGCGCCGCGTTCCAGTTGCGAGG
TGTTAGCTACTACGCAATGGAGGCTGCAGCGAGACCGCCACTAGATTTCGGGGCCGGGCTG
GAGTACAGCCCGATCCCCAACACCAAACCCGGGGGCTTGAGGGTTGAAATGACGCTCGAAC
AGGCATGCCCGCCAGAATACTGGCGGGCGCAATGTGCGTTCAAAGATTCGATGATTCACTGA
ATTCTGCAATTCACATTACTTATCGCATTTTGCTGCGTTCTTCATCGATGCCAGAACCAAGAGA

TCCGTTGTTGAAAGTTTTGATTTATTTGTTTTTAGACTCAGAAGTTACACTAAAATCAGAGT
TTTGTGGTTCCTGCGGCGGGCCGTCCCGTTTTACGGGGCGCGGGCTGATCCGCCGAGGCAAC
ATAAAGGTATGTTCACAGGGGTTTGGGAGTTGTAAACTCGGTAATGATCCCTCCGCTGGTTC
ACCAACGGAGACCTTGTTACGACTTTTACTTCCTCTAAATGACCGAGTTTGGAGAGCTTTCC
GGCCCTGAGCGGTAGTTGCCCACCACTCTGGGCCAGTCCGGACGCCTCACTGAGCCATTCAA
TCGGTAGTAGCGACGGGCGGTGTGTACAATAGGGGGGGAGCATATAAAAGC

CHS_2 TEF-1α sequence:

GACTCACCTTAACGTCGTCGTCATCGGCCACGTCGACTCTGGCAAGTCGACCACTGTGA
GTACTCTCCTCGACAATGAGCTTATCTGCCATTGTCAATCCCGACCAAGACCTGGCGGGGTAT
TTCTCAAAGTCAACATACTGACATCGTTTCACAGACCGGTCACTTGATCTACCAGTGCGGTG
GTATCGATAAGCGAACCATCGAGAAGTTCGAGAAGGTTAGTCACTTTCCCTTCGATCGCGCG
TCCTTTGCCCATCGATTTCCCCTACGACTCGAAACGTGCCCGCTACCCCGCTCGAGACCAAA
AATTTTGCAATATGACCGTAATTTTTTTTGGTGGGGCACTTACCCCGCCACTTGAGCGACGGG
AGCGTTTGCCCTCTTTCCATTCTCACAACCTCGATGAGTGCGTCGTCACGTGTCAAGCAGTC
ACTAACCATTCAACAATAGGAAGCCGCTGAGCTCGGTAAGGGTTCCTTCAAGTACGCCTGGG
TTCTTGACAAGCTCAAGGCCGAGCGTGAGCGTGGTATCACCATCGATATTGCTCTCTGGAAG
TTCGAGACTCCTCGCTACTATGTCACCGTCATTGGTATGTTGTCGCTCATGCTTCATTCTACTT
CTCTTCGTACTAACATATCACTCAGACGCTCCCGGTCACCGTGATTTCATCAAGAACATGATC
A

CHS_3 ITS sequence:

TTGGAGCGCTCGAGATTTGGCATCCACCAGACGGGGCTCTCACCCTCTATGGCGTCCCG
TTCCAGGGAACTCGGAAGGCACCGCATCAAAAGTATCCTCTACAAATTACAACTCGGGCCCG

AAAGCCAGATTTCAAATTTGAGCTGTTGCCGCTTCACTCGCCGTTACTAGGGCAATCCCTGTT

GGTTTCTTTTCCTCCGCTTATTGATATGCTTAAGTTCAGCGGGTATTCCTACCTGATCCGAGGT

CAACATTCAGAAGTTGGGGTTTTACGGCATGGCCGCGCCGCGTTCCAGTTGCGAGGTGTTAG

CTACTACGCAATGGAGGCTGCAGCGAGACCGCCAATGTATTTCGGGGGCGGCACCGCCCAG

AAGGGCAGAGCCGATCCCCAACACCAAACCCGGGGGCTTGAGGGTTGAAATGACGCTCGA

ACAGGCATGCCCGCCGGAATACCAGCGGGCGCAATGTGCGTTCAAAGATTCGATGATTCACT

GAATTCTGCAATTCACATTACTTATCGCATTTTGCTGCGTTCTTCATCGATGCCAGAACCAAG

AGATCCGTTGTTGAAAGTTTTGATTTATTTGTTTGTTTTACTCAGAAGTTACAATAAGAAACA

TTAGAGTTTGGGTCCTCTGGCGGGCCGTCCCGTTTTACGGGGCGCGGGCTGATCCGCCGAGG

CAACATTAAGGTATGTTCACAGGGGTTTGGGAGTTGTAAACTCGGTAATGATCCCTCCGCTG

GTTCACCAACGGAGACCTTGTTACGACTTTTACTTCCTCTAAATGACCGAGTTTGGAGAGCT

TTCCGGCCCTGAGTGGTAGTTGCCCACCTCTCTGGGCCAGTCCGGACGCCTCACTGAGCCAT

TCAATCGGTAGTAGCGACGGGCGGTGTGTACATGGG

CHS_3 TEF-1α sequence:

TGGCAAGTCGACCACTGTAAGTACAACCAACAGCGGGTTGCTTATCTGCACTCGGAAT

CCGCCAAACCTGGCGGGGTATCACCAAAACATCTTGCTAACTTTTGACAGACCGGTCACTT

GATCTACCAGTGCGGTGGTATCGACAAGCGAACCATCGAGAAGTTCGAGAAGGTTAGTCA

ATATCCCTTCGATTACGCGCGCTCCCATCGATTCCCACGATTCGCTCCCTCACTCGAAACAC

ATCCATTACCCCGCTCGAGTCCGAAAATTTTGCGGTGCGACCGTGATTTTTTCTGGTGGGGT

ATCTTACCCCGCCACTCGAGTCACGGATGCGCTTGCCCTGTTCCCACAAAACCTTACCACCC

TGTCGCGCACTACATGTCTTGCAGTCACTAACCACTGGACAATAGGAAGCCGCCGAGCTCG

GAAAGGGTTCCTTCAAGTACGCCTGGGTTCTTGACAAGCTCAAAGCCGAGCGTGAGCGTGG

TATCACCATTGATATCGCTCTCTGGAAGTTCGAGACTCCTCGCTACTATGTCACCGTCATTC

GGTATGTTGTCACTGTCTCGACACTATCATGTGATTCATCATGCTAACATCTCTCAGATGCC

CCCGGTCATCGTGATTTCATCAAGAACATG
